# Feasibility of a specific task-oriented training versus its combination with manual therapy on balance and mobility in people post stroke at the chronic stage: study protocol for a pilot randomised controlled trial

**DOI:** 10.1186/s40814-021-00886-0

**Published:** 2021-07-27

**Authors:** Kristina Traxler, Franz Schinabeck, Eva Baum, Edith Klotz, Barbara Seebacher

**Affiliations:** 1 Therapiezentrum “Kinema”, Neukirchen b. hl. Blut, Germany; 2 Department for Health Sciences, Medicine and Research, Faculty of Health and Medicine, Danube University Krems, Krems an der Donau, Austria; 3 Überörtliche Gemeinschaftspraxis Hohenwarth/ Lam, Hohenwarth, Germany; 4 Praxis Mittelpunkt Mensch, Furth im Wald, Germany; 5 Clinical Department of Neurology, Medical University of Innsbruck, Innsbruck, Austria; 6 VASCAGE GmbH, Research Centre on Vascular Ageing and Stroke, Innsbruck, Austria

**Keywords:** Stroke, Neurological physiotherapy, Manual therapy, Task-oriented training, Postural balance, Walking, Mobility limitation, Range of motion, Ankle joint

## Abstract

**Background:**

Large studies have shown that stroke is among the most relevant causes of acquired adult disability. Walking and balance impairment in stroke survivors often contribute to a restriction in daily activities and social participation. Task-oriented training (TOT) is an effective treatment strategy and manual therapy (MT) is used successfully to enhance ankle joint flexibility in this population. No study, however, has compared TOT against its combination with MT in a randomised controlled trial. Aims of this pilot study are therefore to explore the feasibility of a full-scale RCT using predefined feasibility criteria. Secondary aims are to explore the preliminary effects of specific TOT with a combined specific TOT-MT versus a control group in people post stroke.

**Methods:**

This is a protocol of a 4-week prospective randomised controlled parallel pilot trial in people post stroke at the chronic stage with limited upper ankle joint mobility and an impairment in balance and mobility. At a German outpatient therapy centre using 1:1:1 allocation, 36 patients will be randomised into one of three groups: 15-min talocrural joint MT plus 30-min specific TOT (group A), 45-min specific TOT (group B), and controls (group C). Training will be goal-oriented including tasks that are based on daily activities and increased in difficulty utilising predefined progression criteria based on patients’ skill levels. Interventions will be provided face-to-face 2 times per week, for 4 weeks, in addition to 20-min concurrent x4 weekly home-based training sessions. Data will be collected by blinded assessors at baseline, post-intervention and 4-week follow-up. The primary outcome will be feasibility assessed by recruitment, retention and adherence rates, compliance, adverse events, falls and the acceptability of the intervention. Secondary outcomes will be walking speed, single and dual tasking functional mobility, ankle range of motion, disability and health-related quality of life.

**Discussion:**

Feasibility provided, results from this study will be used to calculate the sample size of a larger randomised controlled trial to investigate the effects of specific TOT and specific TOT-MT compared to a post stroke control group.

**Trial registration:**

German Clinical Trials Register, DRKS00023068. Registered on 21.09.2020, https://www.drks.de/drks_web/navigate.do?navigationId=trial.HTML&TRIAL_ID=DRKS00023068.

**Supplementary Information:**

The online version contains supplementary material available at 10.1186/s40814-021-00886-0.

## Introduction

Walking and balance impairment contribute to functional disability experienced by many stroke survivors, frequently affecting social participation [1]. Postural control deficits in the stroke population are related to reduced walking speed and dynamic mobility, greater gait variability and fall rates [2]. Functional walking limitation and motor control deficits in people post stroke are associated with the upper motor neuron syndrome [3]. Foot drop is often observed in stroke patients, induced by spasticity, weakness of the foot dorsiflexors, shortening of plantarflexors and ankle range of motion (ROM) limitation [4, 5]. Such a complex movement disorder requires differentiated therapy strategies, to address both the structural and functional movement limitations. Upper ankle joint, i.e. talocrural manual therapy (MT) [6] and MT with movement, leads to increased joint ROM alongside balance improvements [7, 8].

Few studies have investigated the effects of talocrural mobilisation and conventional functional training in chronic stroke patients, showing conflicting results. Kluding and Santos compared the effects of functional practice with ankle joint mobilisations against functional practice alone [9]. Study results showed increased ROM of the ankle joint after MT. Improved weight transfer to the paretic side during sit-to-stand was seen after functional training whereas combined practice impaired weight transfer. An and Jo explored active talocrural mobilisation as an add-on to conventional physiotherapy in chronic stroke patients. In contrast to the aforementioned study, improvements in ankle ROM, strength, mobility and weight transfer to the paretic side were observed post-intervention [10].

From a neurological physiotherapy and motor learning perspective, it appears relevant practising meaningful tasks to enhance motor control and dynamic mobility. Specific strategies are needed to promote motor learning and long-term improvement in motor function [11]. Task-oriented training (TOT) is established in stroke rehabilitation, including a variety of trained activities and progression strategies [12–14] where patients actively interact with the environment striving for a defined and meaningful goal [15]. While the training is individually targeted, progression should be predefined. Patients will be invited to choose training tasks according to their preference and activities of daily living (ADL). In addition, effective training programmes involve a specific, outstanding, challenging, repetitive, variable and intensive task practice [16–18]. It appears logical that patients who consider the training programme useful and designed for their daily needs engage more intensively. Finally, significant motor learning literature has suggested adopting an external focus of attention during the training, directed to the desired outcome, a device or the environment [19]. Conventional physiotherapy may not meet this criterion without explicit intention.

To our knowledge, only one study has investigated the effects of a specific TOT as compared to a combined wrist joint MT and upper-limb TOT approach [20]. Hand function and joint mobility significantly improved after the combined training than compared to TOT alone. This cross-sectional study did not assess long-term outcomes. A randomised controlled trial (RCT) with a follow-up period is therefore needed which applies the combined intervention protocol to the lower limbs. This pilot study will be conducted to evaluate the feasibility of the methods of a larger trial and to explore the preliminary effects of a specific TOT, versus its combination with talocrural mobilisation on balance, mobility, ankle ROM, number of falls and health-related quality of life (HRQoL) in people post stroke.

The research question is as follows: Is a full-scale RCT feasible to investigate the effects of specific TOT versus its combination with talocrural MT on balance, mobility, ankle ROM, number of falls and HRQoL, based on the feasibility criteria of recruitment, retention and adherence rates, compliance, adverse events, falls and acceptability of the intervention? The null hypothesis is that a larger main study need not be performed.

### Objectives

Explore the feasibility of a larger RCT that will validate the study results and investigate the effects of specific task-oriented mobility and balance training versus its combination with MT on dynamic balance in people at the chronic post stroke stage. Secondary objectives will be to investigate the preliminary effects of the same training on dynamic balance, mobility, walking speed, upper ankle ROM, number of falls, disability and HRQoL.

### Study design and location

**Fig. 1:**
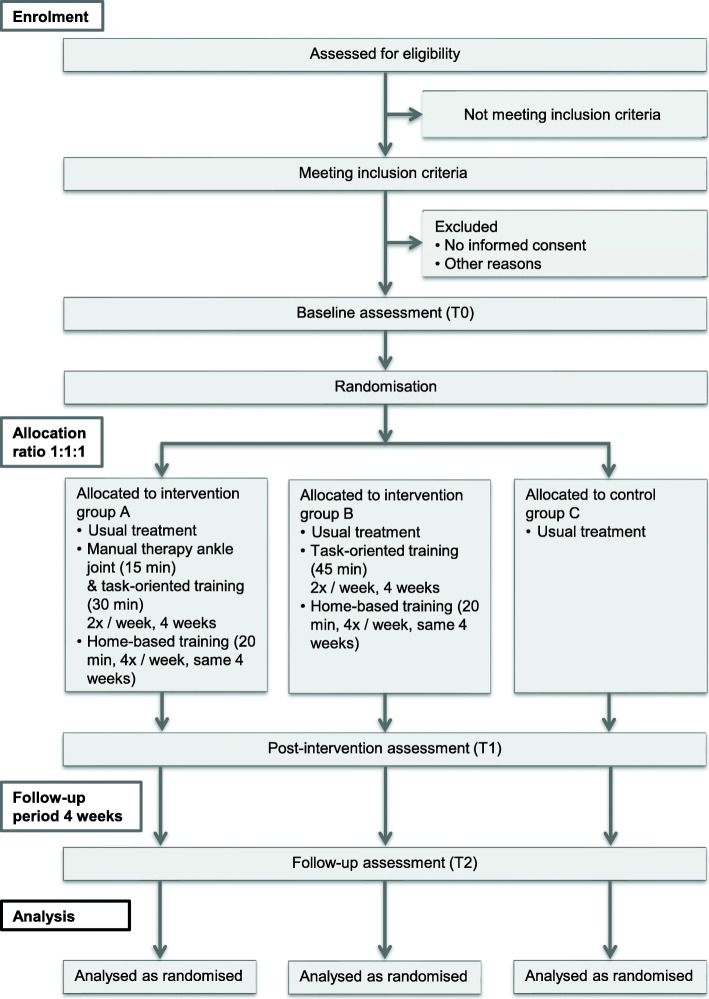
Flow diagram

The supporting CONSORT checklist for this study is available as additional information; see Additional file 1. This will be a prospective randomised controlled pilot trial in chronic stroke patients comprising two parallel intervention groups and a control group. The primary outcome will be feasibility assessed by recruitment, retention and adherence rates, compliance, adverse events, falls and the acceptability of the intervention. Block randomisation will use permuted blocks of 3 and 6 and 1:1:1 allocation. A CONSORT flow diagram is shown in Fig. 1. The study location will be a physiotherapy practice in rural Germany, chosen because the home-based intervention has been designed for stroke patients at the chronic stage who have regained at least a minimum level of mobility.

## Methods

### Patients

#### Inclusion criteria

Patients eligible for the trial must comply with all of the following at randomisation:

1. ≥18 years of age
2. People who have had an ischaemic or haemorraghic stroke at least 6 months prior to study start
3. Limited dorsiflexion ROM in the upper ankle joint on the hemiparetic side: maximum 0° active ROM; maximum 5° of passive ROM
4. Capability to independently transfer from a seating to a standing position without an assistive device—use of the hands is allowed
5. Ability to independently walk without an assistive device for at least 10 m
6. Confirmation by the principle investigator (PI) of the study (medical clinician) that there are no contraindications connected to exposure to active training

#### Exclusion criteria

Patients will be excluded from the study if they meet any of the following criteria:

1. Any contraindication to MT (ankle instability, ankle inflammation or traumas, etc.)
2. Any not fully recovered injuries of the musculoskeletal system
3. Walking impairment due to orthopaedic reasons, e.g., due to previous surgery in the lower extremity region
4. Relevant ROM reduction of the hip or knee joints
5. Any cognitive or communication impairment hindering participation in this pilot study according to the study PI (dementia, severe aphasia, being under legal guardianship, etc.). Screening for cognitive decline will be performed using the Mini Mental State Examination (MMSE) [21], with a score of ≤24 representing cognitive impairment [22]. Cognitive dysfunction will also be evaluated clinically by treating neurologists who refer patients to the study. Dementia will be defined as a patient’s report or the clinical presence of typical symptoms such as impaired thinking, memory, orientation, attention, calculating, reasoning, self-management, language skills, problem solving, visual perception and ability to perform everyday activities.
6. Diabetes mellitus
7. Rheumatic diseases
8. Chronic pain treated by drug therapy
9. History of previous stroke with persistent, impairing neurological symptoms (defined by a Modified Rankin Scale score (mRS) [23] of ≥3)
10. Comorbidity hindering participation, according to the study PI (life expectancy <12 months, cancer, severe heart insufficiency, etc.)
11. mRS score ≥4
12. Pregnancy

#### Recruitment and informed consent

The study will be advertised using recruitment flyers at medical and physiotherapy practices within a 30-km radius of the study location. Written informed consent will be taken by a medical doctor as per German legislation and oral and written information will be provided for the patients by a neurological physiotherapist. Additionally, a trained physiotherapist who holds a Master’s degree in musculoskeletal physiotherapy and is studying for another Master’s degree in neurological physiotherapy (KT) will explain the study intervention in detail. Oral and written information will be given in comprehensible lay language and questions answered regarding study procedures.

#### Randomisation, concealment of allocation and blinding

Randomisation will be stratified on age, the most relevant predictive factor for balance and mobility change in people post stroke (strata: under 70; 70 and over) [24]. Stratification will help ensure similarity between the intervention and control groups regarding age. Mixed randomisation will be performed on the two age groups, consisting of simple and blocked randomisation [25] and applying the following procedure: (1) permuted blocks of three or six are generated with an online random number generator (Sealed Envelope, London, UK); (2) after having randomised a predefined, small and varying number of patients to the three groups, a block size of an interspersed uneven block of 5–11 is chosen, with predefined differences in the sample sizes of the allocated groups; (3) a simple randomisation sequence is generated using the same random number generator; and (4) uneven distribution of As, Bs and Cs provided, another set of permuted blocks of three or six is generated. Details of blocking will be kept in a separate, unavailable document.

Allocation concealment will be performed to avoid allocation bias. Each patient will receive a unique identification number (ID). Based on the randomisation list, sequentially numbered sealed opaque envelopes including group allocation numbers A, B and C will be created for each stratum, given to patients in the order they are recruited. Patients will be asked not to discuss their allocation until study completion. Should a patient nonetheless report details of his or her group allocation, the patient will be informed about the importance of blinding and asked again not to tell anybody about the allocation. In such a case, a second assessor will perform the assessments. The allocation sequence and sealed envelopes will be created by a researcher who is not involved in the study procedures (BS). Patients will be assessed for eligibility and enrolled by the chief investigator (FS) and assigned to the intervention or control groups by the care provider, a physiotherapist-researcher (KT). Outcome assessors (EB; EK) will be blinded to the group allocation of patients. It will not be feasible to blind the care provider.

### Interventions

Eligible patients will be randomised into one of three groups:

- Patients in group A (Gp A) will receive a 15-min talocrural joint MT directed at the dorsiflexion before a 30-min specific task-oriented mobility and balance training 2 times per week. Further, a 20-min home-based ankle mobilisation and TOT programme 4 times per week, for 4 weeks.
- Patients in group B (Gp B) will receive a 45-min specific task-oriented mobility and balance training 2 times per week. Further, a 20-min home-based TOT programme 4 times per week, for 4 weeks.
- Patients in group C (Gp C) will receive no specific treatment.

All patients will receive their usual care involving appointments with the treating neurologist or other medical doctors and any medication such as anticoagulants or antihypertensive drugs. Interventions in groups A and B will be provided face-to-face by an experienced physiotherapist (KT). Patients will be asked to perform the home-based training on the intervention-free days only and given free choice of one rest day per week.

#### Manual therapy

**Fig. 2:**
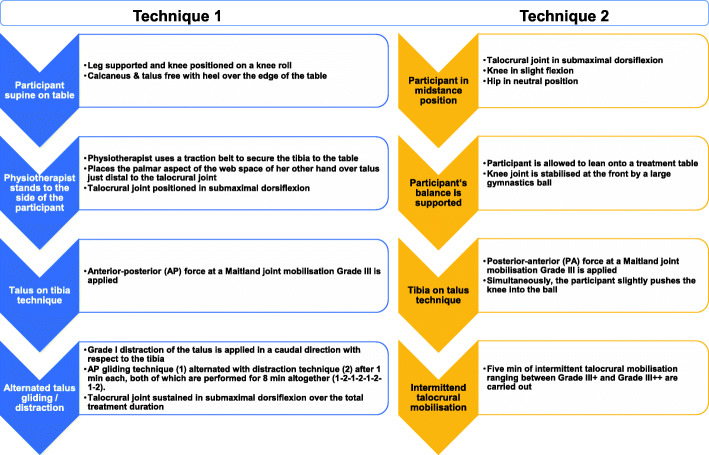
Manual therapy techniques

MT will be carried out by an experienced physiotherapist who holds a Master’s degree in musculoskeletal physiotherapy (KT). MT will be performed according to American Academy of Orthopaedic Manual Physical Therapists guidelines [26] and directed towards the upper ankle joint structures and aim to increase the dorsiflexion ROM. Two different talocrural mobilisation techniques will be used. First, a talus on tibia technique at a Maitland joint mobilisation grade III will be employed on the submaximally dorsiflexed talocrural joint, followed by a grade I tibia distraction, alternated with anterior-posterior gliding. Second, a tibia on talus technique with a posterior-anterior force at a Maitland joint mobilisation grade III will be used on the submaximally dorsiflexed talocrural joint and combined with active forward-movements of the knee joint, followed by intermittent talocrural mobilisation ranging between Maitland grades III+ and III++, all of which are explained in detail in Fig. 2.

#### Face-to-face task-oriented training

TOT will focus on activities involving mobility and dynamic balance. Activities will be based on the requirements of daily living, i.e. avoiding or stepping over obstacles or picking-up objects from the ground. Patients will be timed while being instructed to perform the goal-oriented activities with as much effort and speed as possible. The number of repetitions (or objects moved) will be counted aloud by the physiotherapist researcher as motivation. Additionally, patients will be encouraged to put maximum effort into the TOT, while remaining comfortable. According to established principles of motor learning, exercises should progress in difficulty taking into account the skill level of the task performer and the condition, under which it is performed [27]. Moreover, patients can choose the task order from a predefined list. Between tasks, patients may rest for 45–60 s. Table 1 presents an intervention chart based on the template for intervention description and replication (TIDieR) checklist [28]. Detailed information on the face-to-face and home-based intervention is provided in Additional file 2.

**Table 1:** Intervention chart

Item no.	Item description		
**1 Brief name**	**Group A**	**Group B**	**Group C**
	Upper ankle joint manual therapy (MT) and task-oriented mobility and balance training (TOT)	Task-oriented mobility and balance training (TOT)	Control group: no intervention
**2 Why**	To evaluate the feasibility of the methods of a larger trial and explore the preliminary effects of a specific TOT versus its combination with talocrural MT on balance, mobility, ankle range of motion (ROM), number of falls and health-related quality of life in people post stroke.		
**3 What materials**	- Information sheet on study procedures - Information booklet on home-based TOT programme - Exercise diary including a compliance checklist		
**4 What procedures**	- Usual care		
	- TOT introduction: in lay language; description of the principles - Supervised TOT performance - Home-based TOT		
	- MT introduction: in lay language; description of the principles - MT performance of the talocrural joint - Home-based ankle mobilising exercise		
**5 Who provides**	The intervention will be provided by a trained physiotherapist who holds a Master’s degree in musculoskeletal physiotherapy and is studying for another Master’s degree in neurological physiotherapy		
**6 How**	- TOT and MT introduction and performance: individually and depending on the group patients will be allocated to - Home-based training: individually		
**7 Where**	- TOT and MT introduction and performance: at Outpatient therapy centre “Kinema”, Germany - Home-based training: At patients’ homes		
	17 minutes, 6 times a week, for 4 weeks		
**8 When and how much**	15 min MT plus 30 min TOT, 2 times per week, for 4 weeks	45 min TOT, 2 times per week, for 4 weeks	
	20 min home-based training, 4 times per week, for 4 weeks; on the supervised intervention-free days		
**9 Tailoring**	Same intervention for all patients	Same intervention for all patients	No intervention
**10 Modifications**	No modifications	No modifications	No modifications
**11 How well planned**	- Intervention adherence and compliance will be assessed using a patient diary, during appointments and at post-intervention - Recording in excel sheets will be performed by the instructing researcher (physiotherapist) - The process will be supervised by the academic supervisor.		
**12 How well actual**	- Adherence rates will be reported as median (range) times per week and percentage (95% confidence interval): face-to-face and home-based training - Compliance will be presented narratively (exercise types and order; number of repetitions; problems and deviations from protocol with reasons or explanations)		

#### Home-based training

The same home-based TOT programme will be used for patients in groups A and B. Patients will be informed they may select the TOT type and order but should alternate tasks over the intervention period. Selected tasks (T) should be performed for 2 min each in sets of 3, e.g. (T2+T5+T7+T8)*3. A 1-min pause will be scheduled between the various tasks, but not between sets of the same task. Patients will be trained carefully by the treating physiotherapist and their exercise performance supervised and adapted weekly. Gp A will receive a self-mobilisation technique of the upper ankle joint. Patients will be instructed to commence their training with self-mobilisation. Patients in Gp B will perform another task-oriented exercise. Patients will be instructed to record their training in an exercise diary, e.g. number of repetitions/time period. Similar to face-to-face training, training will progress in difficulty (Additional file 2).

#### Strategies to improve adherence to interventions

A 4-week goal-oriented face-to-face intervention will be used. Goal setting and supervision of exercises by a physiotherapist have been shown to facilitate adherence [29]. The study intervention will be based on ADL relevant to people post stroke which may enhance adherence. In addition, the described TOT could be motivating for patients. The 1:1 treatment sessions will be scheduled at the patients’ convenience.

As for home-based training, simple exercise instructions written in lay language and including photos will be employed to guide and improve training adherence. Patients will be allowed to freely choose the time, task order and type; however, all tasks should be trained. Tasks will be demonstrated and explained by the physiotherapist. The scheduled duration of the home-based training is 20 min, likely perceived doable by patients. An exercise diary with a compliance checklist will be used to monitor adherence and checked by the physiotherapist weekly throughout the intervention period. Patients will be reminded of their post-intervention and follow-up appointments.

### Data collection

Demographic (gender, age) and stroke-specific data (lesion side; disease duration; modified Rankin scale score) will be extracted from patients’ charts at eligibility screening (−*t*_1_). Study-specific assessment data will be collected at baseline (*t*_1_), post-intervention (*t*_2_) and follow-up (*t*_3_) at 4 weeks after completion (Table 3) by two physiotherapists blinded to the study intervention. Assessments will be performed at similar times to increase comparability. In addition to ROM measurement, the validated German version of all assessments will be used. Semi-structured interviews will gather in-depth information regarding acceptance of the study intervention. Changes from baseline and from post-intervention will be evaluated for all outcomes using means, medians, proportions and text as appropriate.

#### Primary outcome and outcome measures

##### Feasibility

- *Recruitment, retention and adherence rates*

The feasibility of conducting a full-scale RCT will be explored using predefined criteria, recording monthly recruitment and adherence rates to the face-to-face intervention. Using an exercise diary, adherence to the home exercise programme and any non-adherence will be recorded, including reasons (e.g. discontinuation due to lack of interest or injury). Any non-retention will also be recorded with reasons (e.g. consent withdrawn) (Fig. 1).

The criteria for feasibility success will be:

- a target recruitment rate of 25% of 145 eligible patients (or 6 patients per month). The number of 145 was estimated according to information received from neurologists and clinicians practising within 30 km of the study centre, concerning the number of eligible chronic stroke patients they have treated within the previous 9 months,
- a target retention rate of 85%,
- a target minimum adherence rate of 75% for face-to-face interventions (6 practice sessions out of a maximum of 8) and
- a target minimum adherence rate of 75% for home-based interventions (12 practice sessions out of a maximum of 16).
  b. *Compliance with the home exercise programme*

An exercise diary will be used to report compliance. Patients in groups A and B will be asked to complete an exercise diary to record type order and repetitions performed of chosen exercises. They should also explain any problems or deviations from the home exercise protocol.

c. *Adverse events and falls*

Falls are considered a relevant outcome from a mobility and balance intervention study because of the associated injury risk. Any adverse events and side effects will be systematically recorded and evaluated using a formal log including the type of adverse event, dates, severity, causality differentiated into serious/non-serious, actions to restore or improve the patient’s wellbeing and outcome of the event. A falls log will be provided to patients, which will be collected at *t*_2_ and *t*_3_, in addition to the number of and reasons for falls within prior 6 months. Study-related adverse events such as training-related injuries will be treated and paid for. Severe adverse events will lead to early study termination.

d. *Acceptability of the intervention*

Acceptability of the intervention will be assessed using semi-structured qualitative interviews comprising four predefined questions presented in Table 2, utilised to learn how the interventions worked for the patients and how well they were appreciated. This is of clinical relevance because high acceptability may help increase motivation, autonomy and adherence in patients.

**Table 2:** Semi-structured interview guide

Introduction	
Main part	
Question #1	Would you please describe your experiences with the training in this study?
Question #2	Is there anything that would motivate you to further participate in this training (i.e. after the study)?
Question #3	What do you think about continuing with the home-exercise programme?
Question #4 (Gp A)	How did you experience the manual therapy of your foot?
Question #4 (Gp B)	How was the duration of the training for you?
Prompt #1	Why so?
Prompt #2	Could you describe this in more detail?
Thanks and conclusion	Many thanks for participating in this interview.

Adapted from [30, 31]

#### Key secondary outcome and outcome measures

##### Dynamic balance (Mini Balance Evaluation Systems Test, Mini BESTest)

The primary outcome will be dynamic balance as assessed by the Mini Balance Evaluation Systems Test (BESTest) [32]. Fourteen items are rated on a 3-point ordinal scale (from 0 = severe to 2 = normal). A transformation table from raw into interval scores is available since the Mini BESTest demonstrated excellent construct validity, unidimensionality, minimal ceiling and no floor effects [32] and a minimal detectable change of 3.0 points [33]. The 4 subscales are transitions/anticipatory postural control, reactive postural control, sensory orientation and stability in gait. Preliminary changes in the key secondary outcome will be used to calculate the sample size for the main trial.

#### Other secondary outcomes and outcome measures

Patients may use their walking aid for all walking tests, but this needs to be documented and used consistently for all trials.

- *Walking speed (10-m walk test, 10MWT)*

The 10MWT at maximum speed will be used as a functional measure of walking. It has been recommended by various task forces [34, 35]. Academy of Neurologic Physical Therapy test instructions will be followed [36] and data recorded in m/s. The average will be calculated from two trials.

For the 10MWT, an excellent concurrent validity has been shown with ADL [37]. Good convergent validity with the 6-min walk test [38] has been observed, an excellent interrater and test-retest reliability and a minimal detectable change (MDC) of 16% [39].

b. *Functional mobility (Timed Up and Go, TUG)*

The TUG [40] is a measure of functional mobility able to detect change over time in patients post stroke [39]. The patient is observed and timed while he or she rises from a chair with armrests, walks 3 m as quickly and safely as possible towards a marking, turns, walks back and sits. Excellent interrater reliability has been found, plus good convergent validity with relevant measures [40]. The MDC of the TUG in stroke patients is 23% [39].

c. *Functional mobility with dual tasking (TUG*_*manual*_ *and TUG*_*cognitive*_*)*

Dual tasking will be assessed using the TUG_manual_ and TUG_cognitive_ where the TUG is combined with a motor (carrying) or cognitive task (subtracting in threes) [41]. For both dual-tasking TUGs, a good test-retest reliability and convergent validity have been confirmed [42, 43]. An MDC of 3.53 s for the TUG_manual_ [42] has been observed in people after stroke. The time difference between the TUG and TUG_cognitive_ is indicative of an increased fall risk [44].

d. *Ankle range of motion (passive and active dorsiflexion and plantarflexion ROM)*

Ankle mobility will be assessed using different techniques to assess the effects of MT.

1. *Non-weight-bearing dorsiflexion and plantarflexion ROM measurement*

Passive and active talocrural dorsiflexion (DF) and plantarflexion (PF) ROM will be assessed in non-weight-bearing supine position with the knees on a standard knee roll (20° flexed), to avoid tensioning of the gastrocnemius muscle [45]. A standard measurement technique is described in detail in Additional file 3. Data will be recorded using the neutral-zero method [46]. Excellent interrater reliability has been demonstrated for ankle dorsiflexion [45] and plantarflexion measures [47].

2. *Weight-bearing dorsiflexion ROM measurement*

Ankle dorsiflexion ROM will also be assessed utilising a tape measure and a weight-bearing lunge facing a wall, as validated by Konor et al. (2012) and outlined in detail in Additional file 3 [48]. Excellent interrater [49] and intrarater reliability, low measurement error and an MDC of 1.1–1.5 cm has been demonstrated for this measurement technique [48].

e. *Disability and HRQoL after stroke (Stroke Impact Scale, version 2.0, SIS v2.0)*

The validated German version [50] of the SIS v2.0 [51] will be used to assess disability and HRQoL. The SIS is a stroke-specific PROM comprising 64 items and assessing 8 domains (strength, memory, emotion, communication, ADL and instrumented ADL (IADL), mobility, hand function, and social participation). A summary score is not calculated, but a physical domain score can be created from the strength, hand function, mobility and ADL/IADL domains. In the emotion domain, items 3a-e and 3g are reverse scored, with all items scored on a 5-point Likert scale. Scores for each domain are transformed to a 0–100 score with lower scores representing poor HRQoL; using the formula: Transformed Scale = [(Actual raw score - lowest possible raw score)/Possible raw score range] × 100. In addition, the SIS v2.0 includes a 0–100 visual analogue scale, with 0 representing no recovery and 100 representing full recovery.

Moderate to excellent internal consistency, test-retest reliability, convergent, divergent and discriminant validity have been demonstrated for the SISv2 [51].

#### Patient timeline

The planned study duration is from 11 September 2020 to 31 October 2021. A detailed overview of the patient timeline is shown in Table 3.

**Table 3:**
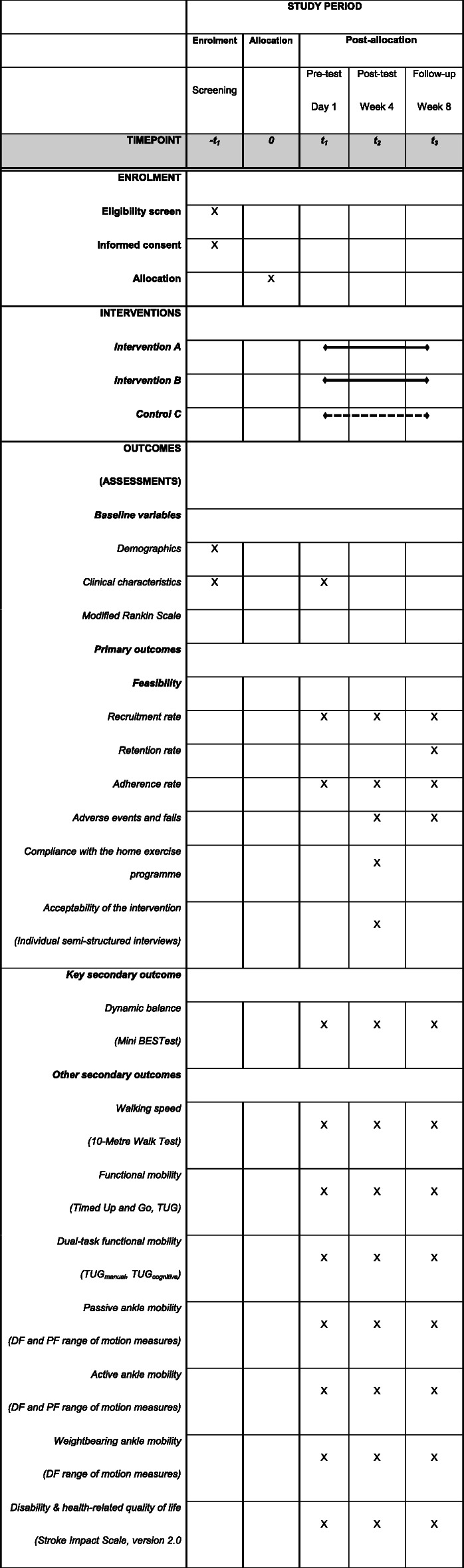
Schedule of enrolment, intervention and data collection

*Mini BESTest* Mini Balance Evaluation Systems Test, *DF* dorsiflexion, *PF* plantarflexion

#### Sample size

The study will be a pilot RCT to provide information for a subsequent larger RCT. The sample size was based on specific objectives of the study such as feasibility, acceptability and falls rather than on estimated effects of interventions. Julious recommends a minimum sample size of 12 patients per group for pilot studies [52]. Therefore, 36 people post stroke will be recruited into three groups (A, B and C). Taking into account an attrition rate of 20% and using the equation $N=\frac{n}{\left(1-\frac{z}{100}\right)}$ where *n* is the sample size of 36 patients and *z* is the expected attrition rate [53], this number may increase to a total of 45 patients. For the sample size estimation of the main trial, imprecision of the standard deviation in small samples will be taken into account [54].

### Data management and data protection

Data quality will be promoted by duplicate (10MWT) or triple measurements (ankle ROM measures, utilising two different measurement approaches). Only the PI and care provider will be aware of the demographic data and have access to the pseudonymisation documents. Double data entry will be performed. Data will be verified by conducting spot checks of value ranges and field types for certain data fields, and logical checks will also be performed (e.g. the order of visits). When checking value ranges, the fields will be checked for permitted values. Field type checks concern whether the values entered fit the field definition (e.g. numerical fields). Any discrepancies found will be rectified.

For the qualitative analysis of interviews, coding will be performed by two independent coders (KT, BS). Codes will be discussed and if a consensus cannot be reached, a third researcher will provide support (FS). Post interview, the interviewer will review and ascertain that all answers have been understood correctly. Patients will be asked to provide feedback on the findings. For the report, patient quotations will be used, together with patient ID and group allocation.

German legislation states any study records and documents must be stored for a period of 10 years after the conclusion of the clinical study. Personal data will be collected, forwarded, stored and evaluated as part of this clinical study in accordance with the legal provisions of the General Data Protection Regulation (EU) 2016/679 (GDPR) and German Data Protection Act 2018 (BDSG).

### Data analyses

#### Statistical analysis

IBM SPSS Software, release 26.0 (IBM Corporation, Armonk, NY, USA) and GraphPad Prism 8, San Diego, CA, will be used for the data analyses. Statistical significance will be defined as a two-tailed *p*-value <0.05. The percentage of missing data will be recorded. Using Little’s test of missing completely at random (MCAR) [55], the data will be checked, signified by a *p*-value >0.05. An intention-to-treat analysis will be performed.

Descriptive statistics will be used for the baseline demographic variables, primary and secondary outcomes. Continuous data will be checked for normal distribution using the Shapiro-Wilk Test, Q-Q plots and histograms. Raw count (number (*N*), %) will be presented for counted (*N* females and males, *N* falls and *N* adverse events if any), compliance (types and order of home exercises, *N* of repetitions and deviations from the protocol) and nominal data (gender, recruitment, retention and adherence rates). Medians (range; 25th and 75th percentiles) will be reported for ordinal data (Mini BESTest if there are missing data, SIS); mean (95% confidence interval (CI)) will be reported for continuous data (age, Mini BESTest if data can be transformed into interval level, 10MWT, TUG, TUG_manual_, TUG_cognitive_, ankle ROM measures).

The recruitment rate (%) will be estimated by dividing the number of consenting patients by the *N* of eligible patients, multiplied by 100. The retention rate determined by dividing the *N* of patients who completed the study by the *N* of the total sample, times 100. Adherence rates will be calculated by dividing the *N* of TOT sessions (8x; 16x) performed by the patients, divided by the *N* of the scheduled (face-to-face; home-based) training sessions over the 4-week study period, times 100 [56]. The eligibility, recruitment and adherence rates will be calculated with their 95% CI according to the Wilson “score” method cited by Newcombe [57]; when the proportion is close to 0 or 1, a Poisson approximation as described by Brown and colleagues will be used [58].

Inductive statistics will detect any trends related to the intervention efficacy acting as a basis for the sample size calculation for a larger main study. The sample size estimation will be based on a power of 80%, an alpha of 5% and the standardised effect size (Cohen’s *d*). Cohen’s *d* will be calculated using the formula *d* = (mean 1−mean 2)/corrected pooled SD. The corrected pooled SD for 80% of trials will be determined using the formula ${SD}_{corrpool}\sqrt{\left(\frac{SD{1}^2+ SD{2}^2}{2}\right)}\ast 1.293$ [54]. Effect sizes will be estimated with 95% CI using an effect size calculator provided by the Centre for Evaluation and Monitoring at the University of Durham, UK [59].

Baseline group differences will be examined using the chi-square test for nominal data, Kruskal-Wallis test for ordinal data and one-way analysis of variance (ANOVA) for continuous data. If a non-normal data distribution is observed in continuous data, data transformation or non-parametrical tests will be performed, as appropriate. Kruskal-Wallis test will evaluate differences between groups and time points, followed by a Bonferroni correction for multiple comparisons. On continuous data, test assumptions for a mixed design ANOVA will be reviewed, with standard correction procedures employed appropriately. In order to evaluate the effects of the intervention on walking speed, mobility, dual-task mobility and ankle ROM, a 2-way mixed design ANOVA will be used, with “time” (*t*_1_, *t*_2_ and *t*_3_) as within-subject factor and “group” (A, B and C) as between-subject factor. If the overall group-by-time interaction is statistically significant, post hoc tests with Bonferroni correction will be applied to determine group differences in pairwise comparisons.

#### Qualitative data analysis

Interviews will be transcribed applying the Dresing and Pehl (2017) transcription rules [60] using F4 Transkript software (Dr. Dresing & Pehl GmbH, Marburg, Germany). Data analysis will be conducted with MaxQDA software (VERBI GmbH, Berlin, Germany) adhering to the Consolidated Criteria for Reporting Qualitative Research (COREQ) [61]. Qualitative content analysis will use a systematic and consistent approach to enhance rigour [62–64].

Coding of the total dataset will be performed by two independent researchers to increase intersubjective conformability [65]. Recurring ideas, concepts, themes and words will be identified. A combined inductive and deductive category development approach, based on the material and the research question, will be used. A coding frame where categories are required to be (apparently) unidimensional, mutually exclusive and exhaustive will be developed. Each dimension should capture a different concept of the text. Relevant material will be selected, structured and marked, text sections segmented to identify main and subcategories. Categories will be defined, named and characterised. Subcategory saturation is indicated after one use. A minimum length of a segment is one sentence. Categories will be continuously adapted [64]. A data matrix will be created and descriptive statistics performed, e.g. frequencies. Categories and subcategories will be illustrated using quotations together with a patient ID and group number (A, B or C). Credibility will be further enhanced through a reflective diary for documenting decisions (reflexivity) and peer debriefing of the interviewer to identify biases and assumptions (truth value) [65]. Records will be kept carefully to ensure consistent and transparent data interpretation [66]. Comparisons across accounts will be sought to ensure various perspectives are represented [67].

### Dissemination

Study results will be disseminated at national and international conferences and published in peer-reviewed journals. Patients will be informed about the study findings via lay-language letters.

## Discussion

Walking impairment and falls lead to reduced social participation in many people post stroke. Patients experience limitations in body structure, function, activities and participation as classified by the World Health Organization [68], and consequences are manifold. Therefore, we considered a combined intervention appropriate, addressing functional, structural and activity components. The primary aim will be to evaluate the feasibility of a larger trial. The secondary aim will be to explore the preliminary effects of a specific mobility and balance TOT versus its combination with ankle MT on balance, mobility and falls in people post stroke. Study results will contribute to the stroke rehabilitation literature as the intervention combines traditional MT approaches with specific TOT using a systematic approach. A goal-oriented training will be employed encouraging external focus. Predefined progression criteria will be used to enable a targeted training based on an individual’s skill level and close to the physical performance limits. Using ADL-based tasks, we hope to induce performance gains in patients, all in the chronic stage after stroke. These tasks could be of some relevance to the patients, with a likely transfer into their daily lives occurring. Semi-structured interviews may help to gain insight into patients’ views of the training intervention. Findings from that might initiate a further shaping of the intervention, which could be explored in a follow-up study. Designed for an outpatient setting in a rural area of Germany, the intervention is regarded practicable, low-cost in terms of equipment and readily transferable to other activities. Feasibility provided and effectiveness shown in a main trial, this may facilitate the implementation of the novel treatment also in outpatient rural environments. Study findings could be applied to devise research projects in people with other neurological disorders involving the upper motor neuron, such as multiple sclerosis or traumatic brain injury.

### Study limitations and strengths

We acknowledge that this feasibility study has a small sample size. All results are to be considered preliminary and need to be validated by a larger study. In this study setting, the lack of objective outcome instruments, e.g. motion capture systems, posturography or digital pressure sensing platforms, may be limiting. Notwithstanding, all ROM measurements are based on relevant literature and are clearly described in the study protocol. Standardised instructions of the two assessors will be provided to enhance inter-rater reliability. In addition, validated tests and questionnaires will be used representing gold standard measurements post stroke. Actual falls will be recorded throughout the study and compared against the patient-reported fall rate within the 6 months prior. It is recognised that some patients may not correctly recall their fall rate, but generally, patients consider falls relevant and may therefore remember them. A further limitation to this study may be that the research team will have to rely on patients’ accurate home-training performance and self-reports of compliance. However, patients will be trained carefully by the treating physiotherapist and their exercise performance supervised and adapted weekly. Additionally, patients will be asked to complete an exercise diary that will be checked regularly. A strength of this study could be its rigorous design, inclusion of relevant reporting checklists and main focus on feasibility as suggested for pilot studies [69]. Another strength seems to be the mixed methods approach as for example, findings from the qualitative analysis might help to identify individual reasons for a lack of acceptability. Finally, if feasibility was shown, the study design would allow to calculate the sample size for a main trial.

### Trial status

The protocol published here is version 1.3 dated 02 August 2020. Recruitment was significantly delayed due to the COVID-19 pandemic. The trial began recruitment on 01 April 2021 and is expected to continue until 31 October 2021.

## Supplementary Information

**Additional file 1.** CONSORT checklist - extension to randomised pilot and feasibility trials

**Additional file 2.** Detailed description of the study intervention

**Additional file 3.** Ankle range of motion measurement

## Data Availability

Datasets generated and/or analysed during the current study will be available from the corresponding author on reasonable request.

## Abbreviations

ADL: Activities of daily living
ANOVA: Analysis of variance
AP: Anterior-posterior
BDSG: German Data Protection Act
Mini BESTest: Balance Evaluation Systems Test, short version
CI: Confidence interval
CONSORT: Consolidated Standards of Reporting Statement
COREQ: Consolidated Criteria for Reporting Qualitative Research
CRF: Case report form
DF: Dorsiflexion
*η*^2^: Partial Eta-squared
GDPR: General Data Protection Regulation
Gp: Group
HRQoL: Health-related quality of life
IADL: Instrumented activities of daily living
ID: Identification number
MCAR: Missing completely at random
MDC: Minimum detectable change
mRS: Modified Rankin Scale
MT: Manual therapy
N: Number
PA: Posterior-anterior
PF: Plantarflexion
PI: Principle investigator
RCT: Randomised controlled trial
ROM: Range of motion
SD: Standard deviation
SIS v2.0: Stroke Impact Scale, version 2.0
*t*: Test
TOT: Task-oriented training
TUG: Timed Up and Go
TUG_cognitive_: TUG with cognitive dual tasking
TUG_manual_: TUG with motor dual tasking
10MWT: 10-m walk test
