# Supplementary material for: Feasibility of a specific task-oriented training versus its combination with manual therapy on balance and mobility in people post stroke at the chronic stage: study protocol for a pilot randomised controlled trial
Source: Pilot Feasibility Stud. 2021 Jul 27;7:146. doi: 10.1186/s40814-021-00886-0 (PMC8313417; doi:10.1186/s40814-021-00886-0)
Supplement: Supplementary file 2 — Additional file 2. Detailed description of the study intervention [file 40814_2021_886_MOESM2_ESM.pdf]

Additional file 2 Detailed description of the study intervention

Face-to-face task-oriented training

Tasks to practise will involve:

- **Task 1:** Weight shifting onto the hemiparetic leg: the non-paretic foot is placed on a footstool in front of the patient; who is asked to move small sandbags from one side of a treatment table in front of them to another using their hand(s). Goal is to move as many bags as possible. After a successful transport of 20 bags, the distance between the patient and table is increased.
- **Task 2:** Weight shifting onto the hemiparetic leg: the patient is asked to step over tensioned rubber bands with her/his non-paretic leg (forth and back). The rubber band, adjusted according to the practictioner's skill level will be as high as possible. Goal is to step over the band as frequently as possible. Initially, the patient is allowed to slightly touch a treatment table. After a successful performance of 20 steps; then, the band height is increased in 5 cm increments.
- **Task 3:** Weight shifting onto the hemiparetic leg (a variation on exercise 2): prior to the training, maximum step length is measured and the floor is marked accordingly. Then, the rubber band is tensioned. The patient should aim to reach the marking with his non-paretic shoe tip when stepping over the rubber band. Goal 1 is to reach the marking. Goal 2 is to perform as many repetitions within a predefined time period (e.g., 1 min). After a successful performance of 20 repetitions, the height of the rubber band is increased by 5 cm.
- **Task 4:** Paretic foot activity during swing phase of gait: Tasks 2 and 3 are performed with the paretic foot. Progression is performed as described for Tasks 2 and 3.
- **Task 5:** Mobility and dynamic balance: Starting from a seated position on a chair without armrests, the patient is asked to collect a small sandbag from a table at a 5-metre distance. Differently sized obstacles are placed on the floor, which need to be stepped over to reach the table. Goal is to collect as many bags as possible. After 3 successful laps, larger objects are used.
- **Task 6:** Mobility and dynamic balance (a variation on exercise 5): The patient is asked to collect a small sand bag from the table, but needs to walk the "narrow track" where the obstacle-covered ground is narrowed by ropes. Goal is to collect as many bags as possible,

using larger objects after 3 successful laps.

- **Task 7:** Dynamic balance: The patient stands on a mini-trampoline (safeguarded by the physiotherapist) and is asked to start swinging. In response to a ‘stop’ command after around 5 seconds, the patient should stop with as few balancing steps as possible. These are counted aloud to the patient who is also informed about previous trials. The numbers of successful trials and of balancing steps within a 3-minute period are recorded. After a successful performance for 1 minute, swinging is replaced by low-effort jumping.
- **Task 8:** Mobility and dynamic balance: Starting from a seated position on a chair without armrests, the patient is asked to collect small, middle-sized and large balls from a table at a 5-metre distance. Cones are placed on the floor, which need to be circumvented using a slalom style. On their way back (no slalom), the patient is asked to transport one ball with both (preferable) or one hand collecting as many (large) balls as possible within a predefined time period (e.g., 3 minutes). After each successful performance of 1 minute, the distance between the cones is reduced by 15 cm.
- **Task 8:** Mobility and dynamic balance: The patient stands on a marked place, the ‘starting point’. A cone with therapy rings placed around them is positioned on the ground within reaching distance of the patient. A number of cones are distributed on the floor. The patient is asked to place one ring around each cone at a time the goal to place as many rings as possible around the cones within a predefined time period (e.g., 3 minutes). After each successful performance of 1 minute, the distance between the cones is reduced by 0.5 metre.

Homebased training

Self-mobilisation of the talocrural joint

- The starting position is a standing forward lunge with the paretic leg back, approximately 1.5 metres apart from a wall and faced towards it.
- The non-paretic leg in the front is flexed and the other leg is extended.
- Patients are instructed to place a traction belt at ankle height around the lower legs. A small towel is placed beneath the belt to avoid incision of the skin, as the belt needs to be tensioned.
- The task: the practitioner is asked to move the back knee as far as possible towards the wall.

- Foot positioning should be maintained and the knee should not deviate inwards.
- Tensioning of the belt should be kept consistent throughout the exercise except when the knee is moved minimally from the tensioned position (backwards). Alternate tensioning and slight relaxation.
- Duration: 3 x 2 minutes separated by 30 min rest periods

Task-oriented training

Patients will be instructed to record their training in an exercise diary, e.g. repetitions within a given time period. Similar to face-to-face training, the training difficulty will be progressed. Between the various tasks, a 1-minute pause will be scheduled, but not between the different progression levels of the same task.

Task 1:

- The patient is seated on a chair without armrests. A second chair is placed at a distance of 1.5 metres, on which an object is placed.
- The task: The patient is asked to assume a sit-to-stand position repeatedly and touch the object with their hand each time.
- Goal is to achieve as many repetitions as possible without a rest and within a 1-minute period.

Progression 1 (foot positioning): after a successful performance of 20 sit-to-stand tasks, the task is progressed:

(a) Sit-to-stand with a positioning of the feet as preferred by the patient.

(b) Sit-to-stand in step position and free choice of which foot is placed back.

(c) Sit-to-stand in step position, with the alternate foot standing in the back.

(d) Sit-to-stand with an emphasis on the weight distribution on the paretic leg.

Progression 2 (chair height):

(a) Normal; (b) reduced.

Progression 3 (dual tasking):

(a) Combination of the task with a cognitive task: subtracting in threes from 100, 150 or 180.

(b) Combination of the task with motor task: carrying a water filled tumbler in the non-paretic hand.

Task 2:

- The patient is seated on a chair without armrests, with a wall at the side. Five meters apart, a bucket or similar object is put on the ground.

- The patient is asked to walk in a tandem walk (or small step width) towards and around the bucket, return to the chair and sit down.
- Goal is to perform as many laps as possible within a predefined time (e.g., 2 minutes).

Progression 1 (time): after a successful performance of 3 laps:

(a) Increased duration (endurance)

(b) Decreased duration with stable number of laps (maximum speed)

Progression 2 (dual tasking): as above

Task 3:

Same as Task 2, but with backwards walking.

Task 4:

- The patient leans against a wall or closed door with their back and slides downwards to assume a ‘high sitting’ position. The non-paretic leg rests on a footstool in front.
- The task involves the patient placing as many clothes pegs as possible on a clothes horse in front of them. Pegs are placed on a tray with either hand, with the tray being to the very left or right.

Progression 1 (dexterity): after successfully placing 20 pegs, the task is progressed:

(a) Pegs are placed on a tray with the paretic hand, with the tray being to the very left or right of the rack.

(b) Pegs are placed on the line with the paretic hand, to the very left or right of the rack.

Progression 2 (strength)

- The same tasks are performed from a ‘low sitting’ position (leaning against the wall).

Progression 3 (dual tasking)

- Combination of the task with a cognitive task: subtracting from 100, 150 or 180 in steps of 3.

Task 5:

- In a ≥5-metre corridor, buckets or similar objects are placed on the floor at a 1-metre distance from the wall.
- The task involves the patient putting differently sized objects into the buckets, e.g. a towel or lighter.
- After placing an object, the patient should return to the starting point and collect the next

object.

- Goal is to place as many objects within a predefined time (e.g., 2 minutes).

Progression 1 (balance): after successfully placing 20 objects, the task is progressed:

- The same task is performed using a tandem walk.

Progression 2 (balance and strength):

- Buckets are replaced by flat objects such as plates.

Task 6:

- In a ≥5-metre corridor, different household objects of small and medium size are placed on the floor and at a 1-metre distance from the wall, e.g. plastic bowls, towels or books.
- The task involves stepping over the objects without touching them, turning around at the last object, and returning the same way. In the presence of greater balance impairment, the patient should be safeguarded by a caregiver or slightly touch the wall with the fingertips.
- Patients are encouraged to alternate with their legs when stepping over the objects.
- Goal is to perform as many laps as possible within a predefined time (e.g., 2 minutes).

Progression 1 (balance): after successfully stepping over 20 objects, the task is progressed:

(a) The task is performed without assistance.

(b) The task is performed using bigger or higher objects.

(c) The task is performed using a tandem walk (or small step width).

Task 7:

- In a ≥5-metre corridor, the patient stands with his/her back against the wall but without touching.
- The patient is asked to walk to the other end of the corridor and back, performing side steps.
- Goal is to perform as many laps as possible within a predefined time (e.g., 2 minutes).

Progression 1 (balance): after successfully performing 3 laps, the task is progressed:

- The same task is performed using maximum sized side steps.

Progression 2 (strength, ankle ROM):

- The same task is performed with flexed knee joints (heels on the ground) and larger side steps.

Task 8:

- The patient stands with legs parallel and the paretic side is close to a wall without touching.

More body weight should be shifted onto the paretic leg.

- There are four tape markings on the floor: in front, on the right and left sides and at the back of the non-paretic foot (around 0.5 metre from the foot).
- The task involves the patient touching the markings alternately.
- Goal is to touch as many markings as possible within a predefined time (e.g., 2 minutes).

Progression 1 (balance): after successfully touching 20 markings, the task is progressed:

(a) Markings are placed at a greater distance from the non-paretic foot.

(b) A small object is placed between the foot and the markings. The same task is performed stepping over the objects.

(c) The same task is performed with middle-sized objects.

Task 9:

Task 9 is identical to Task 8 except that the paretic leg performs the stepping.
