# Supplementary material for: Feasibility of a specific task-oriented training versus its combination with manual therapy on balance and mobility in people post stroke at the chronic stage: study protocol for a pilot randomised controlled trial
Source: Pilot Feasibility Stud. 2021 Jul 27;7:146. doi: 10.1186/s40814-021-00886-0 (PMC8313417; doi:10.1186/s40814-021-00886-0)
Supplement: Supplementary file 3 — Additional file 3. Ankle range of motion measurement [file 40814_2021_886_MOESM3_ESM.pdf]

Additional file 3 Ankle range of motion measurement

Non-weightbearing dorsiflexion and plantarflexion ROM measurement

Passive and active talocrural dorsiflexion (DF) and plantarflexion (PF) ROM will be assessed in non-weight-bearing supine position with the knees on a standard knee roll (20° flexed), to avoid tensioning of the gastrocnemius muscle (1). The fibula axis (from lateral malleolus to the tip of the fibula head) and lateral foot sole will be marked with pen. Using standardised methods, goniometers have been found valid and reliable for assessing the ROM of joints of the extremities (2). Passive and active dorsiflexion and plantarflexion ROM will be assessed where the therapist and patient will move the foot end of range, respectively. The goniometer fulcrum will be placed over the lateral calcaneus at the bisection of the fibula and fifth metatarsal, the stationary arm parallel to the fibula axis and the movable arm parallel with the foot sole (3). A neutral (zero) position will be defined as the position where the fool sole and fibula axis are at a right angle. Measurement will be performed three times, from which an average will be calculated. Both sides will be assessed to allow for comparisons. Data will be recorded using the neutral-zero method (3).

Weightbearing dorsiflexion ROM measurement

ROM will also be assessed using a tape measure attached to the ground perpendicular to a wall. A weight-bearing lunge facing a wall will be utilised to measure ankle dorsiflexion as validated by Konor et al. (2012) and outlined in detail in Additional File 2 (4). The patient will assume a lunge position with bare feet, with the great toe 10 cm from the wall, heel on the ground and knee in line with the second toe. Touching the wall with two fingers of each hand will be allowed. Patients will be instructed to lunge forward, aiming towards the wall with their knee whilst keeping their heel on the ground. Should it be possible to touch the wall with the knee, the foot will be moved away from the wall in increments of 1 cm and the lunge repeated. The heel and subtalar position will be continuously monitored. Once the heel loses contact with the ground or the knee from the wall, the foot will be moved towards the wall again. This will be done in very small increments to find the optimum position for the measurement (5). Likewise, if the patient is not able to reach the starting position without raising the heel, they will be asked to move their foot towards the wall, observing the outlined principles. Evidence has shown that each centimetre of the distance of the great toe from the wall corresponds to approximately 3.6° of

ankle dorsiflexion (4, 6). The distance from the wall will be measured three times and the average taken.

Additional References

1. Baumbach SF, Brumann M, Binder J, Mutschler W, Regauer M, Polzer H. The influence of knee position on ankle dorsiflexion - a biometric study. BMC musculoskeletal disorders. 2014;15:246.
2. Gajdosik RL, Bohannon RW. Clinical measurement of range of motion. Review of goniometry emphasizing reliability and validity. Phys Ther. 1987;67(12):1867-72.
3. Debrunner HU. Gelenkmessung (Neutral-0-Methode), Längenmessung, Umfangmessung. Bern: Bulletin des Offiziellen Organs der Arbeitsgemeinschaft für Osteosynthesefragen; 1971.
4. Konor MM, Morton S, Eckerson JM, Grindstaff TL. Reliability of three measures of ankle dorsiflexion range of motion. International journal of sports physical therapy. 2012;7(3):279-87.
5. Hoch MC, McKeon PO. Normative range of weight-bearing lunge test performance asymmetry in healthy adults. Manual therapy. 2011;16(5):516-9.
6. Bennell KL, Talbot RC, Wajswelner H, Techovanich W, Kelly DH, Hall AJ. Intra-rater and inter-rater reliability of a weight-bearing lunge measure of ankle dorsiflexion. The Australian journal of physiotherapy. 1998;44(3):175-80.
